# Synthesis and Investigation of Novel Optical Active SiO_2_ Glasses with Entrapped YAG:Ce Synthesized via Sol–Gel Method

**DOI:** 10.3390/gels9060488

**Published:** 2023-06-13

**Authors:** Monika Skruodiene, Meldra Kemere, Greta Inkrataite, Madara Leimane, Rimantas Ramanauskas, Ramunas Skaudzius, Anatolijs Sarakovskis

**Affiliations:** 1 Institute of Solid State Physics, University of Latvia, Kengaraga str. 8, LV-1063 Riga, Latvia; 2 Institute of Chemistry, Faculty of Chemistry and Geosciences, Vilnius University, Naugarduko 24, LT-03225 Vilnius, Lithuania; 3 State Research Institute Center for Physical Sciences and Technology, Saulėtekio av. 3, LT-10257 Vilnius, Lithuania

**Keywords:** optical active, luminescence, sol–gel, synthesis, YAG:Ce, SiO_2_, glass

## Abstract

We present a crack-free optically active SiO_2_ glass-composite material containing YAG:Ce synthesized via a modified sol–gel technique. A glass-composite material consisting of yttrium aluminum garnet doped with Ce^3+^ (YAG:Ce) was entrapped into a SiO_2_ xerogel. This composite material was prepared using a sol–gel technique with modified gelation and a drying process to obtain crack-free optically active SiO_2_ glass. The concentration of the YAG:Ce was from 0.5 to 2.0 wt%. All synthesized samples were characterized via X-ray diffraction (XRD) and scanning electron microscopy (SEM) techniques, confirming their exceptional quality and structural integrity. The luminescence properties of the obtained materials were studied. Overall, the prepared samples’ excellent structural and optical quality makes them great candidates for further investigation, or even potential practical application. Furthermore, boron-doped YAG:Ce glass was synthesized for the first time.

## 1. Introduction

For a long time, sol–gel techniques have been used for the fabrication of glasses and ceramics. Sol–gel synthesis is a widely used method for the preparation of glasses. This method involves the creation of a sol, which converts into a gel and then a glass upon thermal treatment. The sol–gel process can produce glasses with various compositions and structures, making it a versatile and flexible technique. One of the advantages of sol–gel synthesis for glass production is its ability to create complex systems with high surface areas and uniform morphology. The process allows for precise particle size, shape control, and consistent glass properties and performance. In addition to offering a cost-effective and scalable approach to glass production, sol–gel synthesis is a sustainable method with a low cost and environmental impact [1,2,3,4,5]. Due to its significant contributions, the sol–gel method is widely acknowledged as a key technique within the mentioned field. It has demonstrated great success in producing nano powders, encompassing oxide, non-oxide, and composite varieties. Furthermore, the sol–gel process promotes the preparation of highly pure materials [6].

The sol–gel technique of preparing glasses is still being studied. Glasses synthesized via the sol–gel method have attracted much interest because of the possibility of synthesizing glass components, for example, optical fibers, lenses, mirrors, etc. Optically active SiO_2_ glasses are a fascinating class of materials due to their interesting optical properties and potential applications in various fields. A small amount of dopant ions causes the optical activity of SiO_2_ glasses. Optically active SiO_2_ glasses have several potential applications in optics and photonics. For example, they can be used in polarization-sensitive optical devices such as polarizers, waveplates, and optical rotators. They can also be used in sensing applications, where changes in optical activity can be used to detect chemical or biological molecules. Optically active SiO_2_ glasses represent a fascinating and rapidly evolving field of research with significant potential applications in optics, photonics, sensing, and fundamental science. SiO_2_ glasses have proven helpful as host materials for rare-earth ions in solid-state lasers. For several practical reasons, glasses doped with Nd^3+^ have found the most comprehensive application. Among other rare-earth-containing materials, Er^3+^-doped glasses are currently generating much interest as fiber amplifiers in optical communications systems [7,8,9,10].

White LED technology is based on blue LED chips, which were first developed by Nichia Co. in 1991. These chips are coated with yellow-emitting phosphor to create white light. One popular phosphor used for this purpose is Ce^3+^-doped YAG phosphor. This phosphor has been available since the 1960s, but it is considered a cool-light phosphor due to the lack of a red component in its emission spectrum. Its emission spectrum can be shifted by substituting the Y^3+^ or Al^3+^ with different ions. For example, replacing Y^3+^ with rare-earth ions such as Tb^3+^, Gd^3+^, Dy^3+^, La^3+^, etc. can introduce a red-shift, while substituting Al^3+^ with ions like Ga^3+^ or In^3+^ can cause a blue shift in the cerium emission. Co-doping with ions such as Pr^3+^ can also introduce a secondary peak in the red spectral range. White light-emitting diodes (wLEDs) with improved optical properties can be developed by co-doping a YAG:Ce phosphor with rare earth ions. Moreover, adjusting the Ce^3+^ concentration or modifying the process parameters can introduce a slight red shift in the emission spectrum. All this research has led to the development of highly efficient and versatile white LED technology [11,12,13,14,15,16].

Commercially available wLEDs typically combine Y_3_Al_5_O_12_:Ce^3+^ yellow phosphor, an InGaN-based blue-emitting LED chip, and epoxy resin for encapsulation. However, this conventional approach suffers from inadequate red emission, leading to poor color rendering and an improper color temperature. Consequently, the practical applications of these wLEDs in advanced lighting technologies are limited [17,18]. Moreover, phosphor-resin-based LEDs are subject to certain drawbacks. These include resin degradation over time due to current flow and differences in refractive indices between phosphors and epoxy resin, resulting in photon scattering and reduced emission intensity. These issues negatively impact the optical performance of the device, affecting aspects such as color purity and coordination. To overcome these limitations, novel phosphor-in-glass (PiG) color converters have been developed. These converters embed the phosphor material within a glass host through a sintering processes. However, PiG materials have drawbacks, including scattering losses attributed to their porous structure and the potential for reactions between the phosphor material and the glass host [19,20,21]. Based on the reasons above, this study presents a novel PiG composite. The composite is based on cerium-doped YAG and SiO_2_ glass fabricated using the sol–gel method. The sol–gel approach was selected due to its ability to achieve atomic-level mixing of ions, resulting in a homogeneous material at lower temperatures. Silica glass was chosen to prevent any undesirable reactions among the constituents. X-ray diffraction was utilized to ensure the phase purity of the composite. Luminescence properties were investigated to analyze the interface interactions between the phosphor and glass matrix. Finally, SEM analysis was conducted to examine the composite’s morphology and the distribution of the phosphor within the matrix.

## 2. Results and Discussion

### 2.1. X-ray Diffraction

**Figure 1:**
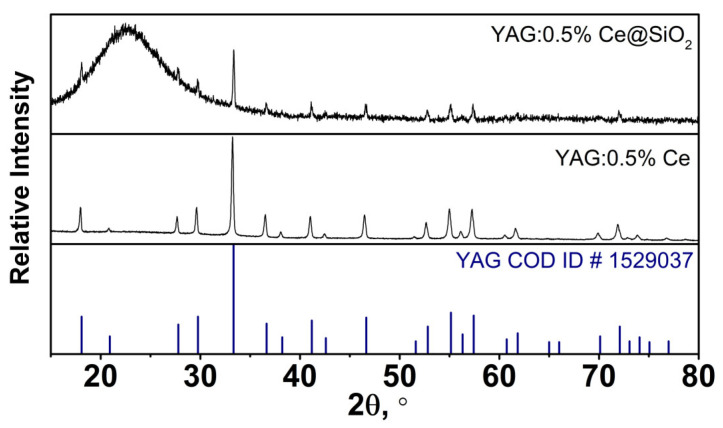
XRD patterns of the 0.5%YAG:0.5%Ce@SiO_2_ composite and YAG:0.5%Ce.

The synthesized samples were analyzed via XRD analysis to determine their composition and purity. All peaks were identified. All reflexes were assigned to the garnet phase (YAG COD ID #1529037). The presence of a broad peak between 20 and 30° serves as a distinctive characteristic of the amorphous SiO_2_, acting as its unique identifier. Notably, there are no shifts observed in the XRD data, which means that no changes were made during the annealing to the pure garnet crystal phase. It can be stated that YAG:Ce@SiO_2_ composite samples are pure. The lattice parameter of doped garnet was refined, which was 12.0234 Å. In comparison with undoped garnet (11.99 Å, COD ID # 4312142) the lattice parameter increased, which confirms the incorporation of larger cerium cations in yttrium positions. The XRD patterns of the YAG:0.5%Ce@SiO_2_ composite and YAG:0.5%Ce are presented in Figure 1.

### 2.2. Scanning Electron Microscopy (SEM) Analysis

**Figure 2:**
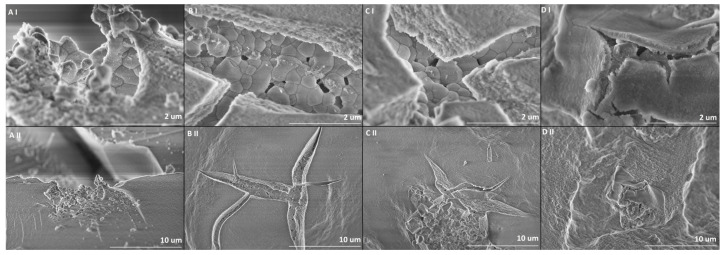
SEM images of the samples in different magnifications. 0.5%YAG:0.5%Ce@SiO_2_ (**A**(**I**,**II**)), 1.0%YAG:0.5%Ce@SiO_2_ (**B**(**I**,**II**)), 1.5%YAG:0.5%Ce@SiO_2_ (**C**(**I**,**II**)) and 2.0%YAG:0.5%Ce@SiO_2_ (**D**(**I**,**II**)).

SEM analysis was performed to determine the surface morphology of the synthesized composites. All synthesized samples were analyzed in different magnifications (I–25 k; II–5 k). Upon examination, it was observed that the surfaces of the samples appeared smooth, albeit with some noticeable microcracks. Within these microcracks, individual garnet particles were visibly present. Notably, these particles exhibited an interconnected structure with irregular shapes. It is worth mentioning that no significant variations were observed among the different samples. Most of the particles are in the size range between 200 to 700 nm. SEM images are presented in Figure 2.

### 2.3. Luminescence Properties

**Figure 3:**
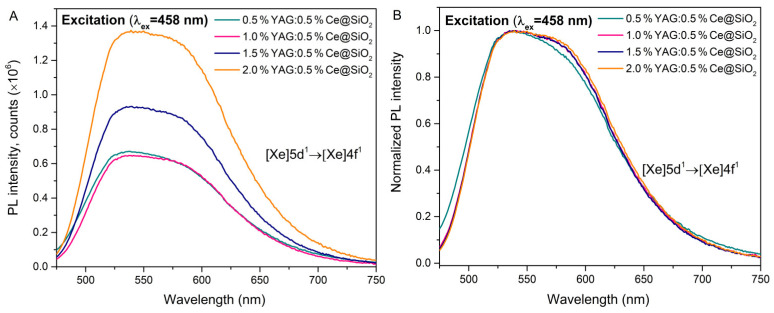
Photoluminescence emission spectra of the samples. Emission spectra of 0.5%YAG:0.5%Ce@SiO_2_, 1.0%YAG:0.5%Ce@SiO_2_, 1.5%YAG:0.5%Ce@SiO_2_ and 2.0%YAG:0.5%Ce@SiO_2_ (**A**), normalized emission spectra of 0.5%YAG:0.5%Ce@SiO_2_, 1.0%YAG:0.5%Ce@SiO_2_, 1.5%YAG:0.5%Ce@SiO_2_ and 2.0%YAG:0.5%Ce@SiO_2_ (**B**).

The emission spectra of synthesized samples excited at 458 nm are depicted in Figure 3. In emission spectra, all samples have a wide band with a peak wavelength (λ_max_ = 538 nm), which is attributed to the [Xe]5d^1^ → [Xe]4f^1^ electron transitions. Increasing the concentration of YAG:0.5%Ce leads to an increase in the emission intensity (Figure 3A). This may be due to an increase in the number of optically active centers in the samples as the concentration increases. Normalized luminescence spectra of YAG:0.5%Ce doped samples are shown (Figure 3B). The spectra exhibit a broad band from around 460 nm to 750 nm, with the maximal intensity at about 538 nm. Notably, the spectrum of the 0.5%YAG:0.5%Ce@SiO_2_ sample is slightly shifted to the shorter emission wavelength. It could be described as coupling the 5d levels with the surrounding crystal field [15,22,23,24,25].

The photoluminescence quantum yield (PLQY) was calculated. The quantum yield of the analyzed samples is shown in Table 1. The highest luminescence quantum yield value (23%) was observed in the samples doped with 1.5% YAG:0.5%Ce and 2% YAG:0.5%Ce. For other samples, it was lower.

**Figure 4:**
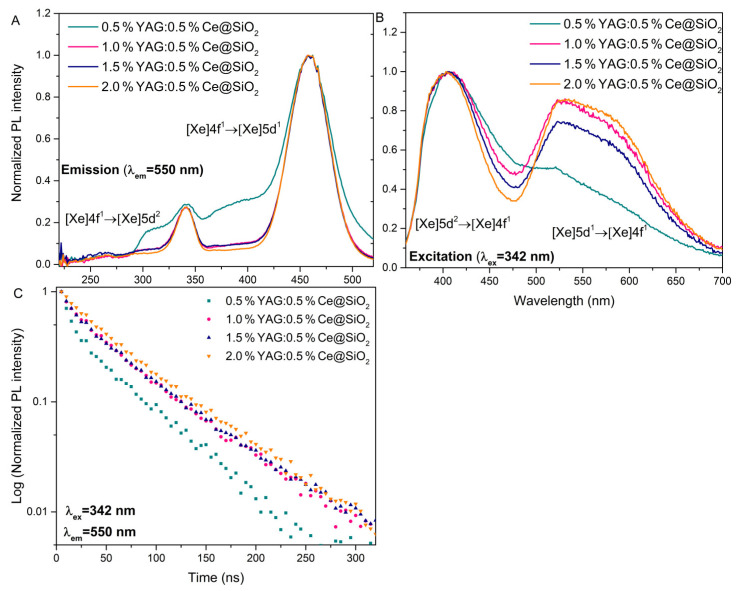
Photoluminescence excitation (**A**), emission (**B**) spectra, and decay curves (**C**) of glass samples.

Another fundamental analysis for optically active glasses is also the decay time measurements. Luminescence properties were further investigated by measuring the photoluminescence decay curves of the synthesized samples. All the analyzed samples exhibit bi-exponential photoluminescence decay. In Figure 4C, luminescence decay kinetics with 342 nm are shown. The luminescence decay of samples doped with 1–2% YAG:0.5%Ce exhibits similar properties, while in a sample with 0.5% YAG:0.5%Ce, the luminescence intensity decreases faster. Excitation and emission (Figure 4A,B) spectra were also measured. Based on the excitation spectra, it is clear that all compounds have wide bands at 342 and 460 nm wavelengths. It can be observed that the most intensive excitation at 342 nm has 0.5%YAG:0.5%Ce@SiO_2_. This could be related to the relative decrease in the green 550 nm luminescence band in the 0.5% YAG:0.5%Ce doped sample (Figure 4B). All the decay curves were calculated and approximated with a double exponential function: *A*_1_ and *A*_2_ are the fitting parameters, *τ*_1_, and *τ*_2_ are decay times of the fast and slow decay components. Using *τ*_1_ and *τ*_2_, the average lifetime was calculated using Equation (1):

(1) $$\langle \tau \rangle =\frac{A_{1}\tau_{1}^{2}+A_{2}\tau_{2}^{2}}{A_{1}\tau_{1}+A_{2}\tau_{2}}$$

The calculated average lifetimes are shown in Table 2. With the increase in the concentration of the YAG:0.5%Ce, the decay time increases. The decay times with the higher concentration are very close to those described in the literature (~60 ns) [26].

## 3. Conclusions

In this study, all single-phase YAG:0.5%Ce garnets synthesized via the sol–gel route were successfully entrapped in SiO_2_ glass. YAG:0.5%Ce@ SiO_2_ xerogel samples were formed with modified gelation and drying to obtain crack-free glass at relatively low temperatures. It was demonstrated that incorporation does not affect the morphology of the analyzed samples. Glass samples contain microcracks with well-shaped irregular sphere-like particles with sizes from 200 to 700 nm. Analyzed glass samples were excited at 458 nm. This showed that increasing the concentration of YAG:0.5%Ce leads to an increase in the emission intensity. The photoluminescence quantum yield (PLQY) was calculated. The highest luminescence quantum yield value (23%) was observed in samples with higher concentrations on YAG:0.5%Ce. Furthermore, by increasing the concentration of the YAG:0.5%Ce, the decay time increases (2.0%YAG:0.5%Ce@SiO_2_ decay time: 60 ns). The aforementioned optical properties indicate that analyzed optically active glasses can be promising candidates for scintillators and white light-emitting diodes.

## 4. Materials and Methods

### 4.1. YAG:Ce Synthesis Procedure via Sol–Gel Route

For synthesized compounds, the following precursors were used: Y_2_O_3_ (99.9% Alfa Aesar, Thermo Fisher (Kandel) GmbH, 76870, Kandel, Germany), Al(NO_3_)_3_·9H_2_O (99.999% Alfa Aesar, Thermo Fisher (Kandel) GmbH, 76870, Kandel, Germany), (NH_4_)_2_Ce(NO_3_)_6_ (99.5% Roth, Carl Roth GmbH & Co. KG, 76187, Karlsruhe, Germany) and H_3_BO_3_ (99.5% ChemPur, ChemPur GmbH, D-76137, Karlsruhe, Germany). Firstly, Y_2_O_3_ was dissolved in a concentrated nitric acid at 80 °C. After that, the nitric acid was evaporated, and the resulting solution was washed with distilled water three times. Every time water was added, the excess water must be evaporated. After washing with distilled H_2_O, 200 mL of distilled water was added. Then, other precursors (Al(NO_3_)_3_·9H_2_O, (NH_4_)_2_Ce(NO_3_)_6_, H_3_BO_3_) were dissolved in the resulting solution. The solution was left to stir on a magnetic stirrer for 2 h at about 50 °C. After 2 h, citric acid was added to the solution in a 1:1 ratio of metal ions and left to stir overnight. Finally, the water evaporated at the same temperature. The resulting gel was dried at a temperature of 400 °C for 24 h. The synthesized xerogel was first heated in air for 2 h at 1000 °C with a 5°/min heating rate, then calcinated in air for 4 h at 1200 °C with a 5°/min heating rate.

### 4.2. YAG:Ce@SiO_2_ Synthesis Procedure via Sol–Gel Route

The samples were synthesized using the sol–gel method following the procedure described by Kajihara [27] with a modified gelation and drying process to obtain crack-free glass. The first step consists of a hydrolysis reaction at room temperature between silicon (Si) organic precursor tetraethoxysilane (TEOS) (99.0% Sigma Aldrich, Sigma-Aldrich Chemie GmbH, 82024 Taufkirchen, Germany) and deionized water, to which a small amount of nitric acid (HNO_3_) (65.0% Sigma Aldrich, Sigma-Aldrich Chemie GmbH, 82024 Taufkirchen, Germany) and a certain amount of YAG:Ce were added. The mixture was then stirred for one hour at room temperature to make a homogeneous solution. The molar ratio of TEOS:H_2_O:HNO_3_ was 1:22:0.002. The sol was with pH value between 1–2. Then, ammonium acetate (AcONH_4_) (97.0% Sigma Aldrich, Sigma-Aldrich Chemie GmbH, 82024 Taufkirchen, Germany) buffer solution in deionized H_2_O was added to the prepared sol to increase the pH value to ~5–6 and stirred for an additional 2 min at room temperature. Prepared sols were then immediately transferred to the container for drying and left for 30 min in the ultrasonic bath to avoid further gel cracking.

Furthermore, the hydrogel was aged slowly in a drying oven starting from room temperature. The temperature was gradually increased to 70 °C. The entire drying process took 168 h. The YAG:Ce@SiO_2_ xerogel samples were further used for the post-treatment process at 1000 °C temperature. A xerogel was placed in a quartz tube, heated at a rate of 5°/min up to the predetermined temperature, and maintained for two hours. Then, translucent YAG:Ce@SiO_2_ glass was obtained.

**Figure 5:**
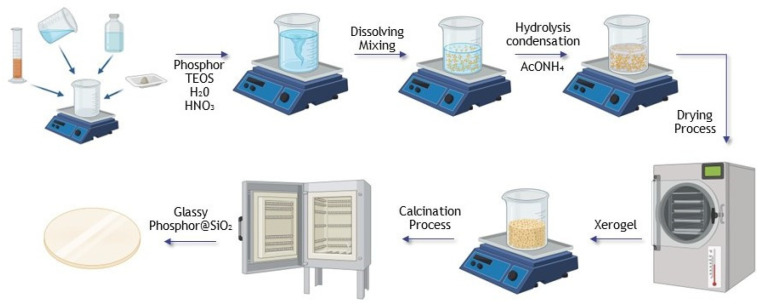
Schematic illustration of the detailed steps of the synthesis route.

A schematic illustration of the detailed steps of the synthesis route of YAG:Ce@SiO_2_ synthesis procedure is presented below (Figure 5).

### 4.3. Characterization

For phase identification at room temperature, the XRD data were collected in 15°–80° 2θ range (step width of 0.01°, scan speed 10°/min, dwell time 5.0 s) using Ni-filtered Cu Kα1 radiation on Rigaku MiniFlexII diffractometer. The measurement current and voltage were set to 15 mA and 30 kV, respectively.

Scanning electron microscopy (SEM) micrographs were taken using Hitachi SU-70 SEM. Powder was fixed on a carbon film. The proper magnification was selected, and images were recorded. The particle size measurements were collected using open-source Fiji (ImageJ 1.52v, Java 1.8.0_112 (64-bit)) software by accidently selecting random particles.

Photoluminescence emission and excitation spectra were recorded at room temperature using a spectrometer from Edinburgh Instruments (model: FLS1000-DD-stm) equipped with a CW 450 W Xenon lamp (model: Xe2) and a cooled red photomultiplier tube (model: R928P) for detection. The spectra were corrected for the instrumental response. Photoluminescence decay kinetics were recorded using a pulsed tunable nanosecond Nd:YAG laser NT 342/3UV from Ekspla. An Andor Technologies spectrometer SR-303i-B and a time-resolved CCD camera DH734-18F-A3 were employed to record photoluminescence decay curves at room temperature.

## Figures and Tables

**Table 1:** The quantum yield of the analyzed glass samples.

Sample Name	PLQY (%)
0.5%YAG:0.5%Ce@SiO_2_	6 ± 0.5
1.0%YAG:0.5%Ce@SiO_2_	12 ± 1
1.5%YAG:0.5%Ce@SiO_2_	23 ± 2
2.0%YAG:0.5%Ce@SiO_2_	23 ± 2

**Table 2:** The calculated luminescence lifetimes and energy transfer efficiency of Ce^3+^ ions in glass samples.

Sample Name	*τ* (ns) ± 3 ns (λ_ex_ = 342 nm, λ_em_ = 550 nm)
0.5%YAG:0.5%Ce@SiO_2_	48
1.0%YAG:0.5%Ce@SiO_2_	53
1.5%YAG:0.5%Ce@SiO_2_	57
2.0%YAG:0.5%Ce@SiO_2_	60

## Data Availability

The data presented in this study are available in [Synthesis and Investigation of Novel Optical Active SiO_2_ glasses with entrapped YAG:Ce synthesized via sol–gel method].
